# Investigating the impact of social media images on users’ sentiments towards sociopolitical events based on deep artificial intelligence

**DOI:** 10.1371/journal.pone.0326936

**Published:** 2025-07-30

**Authors:** Nafiseh Jabbari Tofighi, Reda Alhajj

**Affiliations:** 1 Department of Computer Engineering, Istanbul Medipol University, Istanbul, Turkey; 2 Department of Computer Science, University of Calgary, Alberta, Canada; 3 Department of Health Informatics, University of Southern Denmark, Odense, Denmark; American University of Beirut, LEBANON

## Abstract

This paper presents the findings of the research aimed at investigating the influence of visual content, posted on social media in shaping users’ sentiments towards specific sociopolitical events. The study analyzed various sociopolitical topics by examining posts containing relevant hashtags and keywords, along with their associated images and comments. Using advanced machine learning and deep learning methods for sentiment analysis, textual data were classified to determine the expressed sentiments. Additionally, the correlation between posted visual content and user sentiments has been studied. A particular emphasis was placed on understanding how these visuals impact users’ attitudes toward the events. The research resulted in a comprehensive dataset comprising labeled images and their comments, offering valuable insights into the dynamics of public opinion formation through social media. This study investigates the influence of social media images on user sentiment toward sociopolitical events using deep learning-based sentiment analysis. By analyzing posts from movements such as Black Lives Matter, Women’s March, Climate Change Protests, and Anti-war Demonstrations, we identified a strong correlation between visual content and public sentiment. Our results reveal that Anti-war Demonstrations exhibit the highest correlation (PLCC: 0.709, SROCC: 0.723), while Climate Change Protests display the lowest alignment (PLCC: 0.531, SROCC: 0.611). Overall, the study finds a consistent positive correlation (PLCC range: 0.615–0.709, SROCC: 0.611–0.723) across movements, indicating the significant role of visual content in shaping the public opinion.

## 1 Introduction

The advent of social media has revolutionized the dissemination and consumption of information, providing a powerful platform for user-generated content and public discourse on various sociopolitical issues. This shift has spurred significant interest among researchers in examining how social media content influences public opinion dynamics. Analyzing sociopolitical sentiment on social media offers a unique window into the public’s perceptions and emotional responses to contemporary issues. By leveraging the vast amount of available data, researchers can explore how online discussions reflect and shape public opinion, offering valuable insights into the drivers and trends of sociopolitical sentiment.

Social media sentiment analysis involves several key steps: identifying relevant sociopolitical topics, compiling and categorizing related content, and applying sentiment analysis techniques to quantify the emotional tone of the discourse. Through this process, researchers can gain a deeper understanding of public attitudes and the factors influencing them. Numerous studies have highlighted the potential of social media platforms to analyze sociopolitical sentiment and gauge public opinion on diverse topics. For example, social media data has been used to assess public sentiment toward government policies, capture emotional responses to significant events, and understand attitudes toward marginalized communities.

This research builds on the existing body of work by focusing on the specific influence of visual content, such as images and videos, posted on social media and their role in shaping user sentiments towards sociopolitical events. Visual content is a powerful communication tool, often evoking strong emotional responses and influencing perceptions in ways that textual content alone cannot. By examining how visual content impacts user attitudes and perceptions, this study aims to provide a more nuanced understanding of the role of media in public opinion formation.

This study addresses several key research questions: How does visual content posted on social media impact users’ sentiments towards different sociopolitical events? The research analyzed various sociopolitical events, such as the Black Lives Matter movement, the Women’s March, climate change protests, and Anti-war demonstrations, by examining social media posts containing relevant hashtags and keywords, along with their associated images and comments. Advanced machine learning and deep learning methods were employed for sentiment analysis, enabling the classification of textual data to determine the expressed sentiments. Additionally, the study investigated the correlation between posted visual content and user sentiments, with a particular emphasis on understanding how these visuals impact users’ attitudes toward the events.

The research culminated in a comprehensive dataset comprising labeled images and their comments, providing valuable insights into the dynamics of public opinion formation through social media. The findings demonstrate significant ways in which social media images can shape public sentiment toward sociopolitical events, highlighting the critical role of visual media in the digital age. This dataset serves as a crucial resource for future studies and applications in social media analysis, offering a foundation for continued exploration into the intricate relationship between visual content and public sentiment. By advancing our understanding of these dynamics, this research contributes to the broader discourse on the influence of social media in shaping public opinion and the role of visual content in contemporary sociopolitical communication.

The rest of the paper is organized as follows. The literature review is included in Sect 2. The methodology is described in 3. The conducted experiments and the obtained results are presented in Sect 4. Sect 5 is conclusions.

## 2 Literature review

The field of research on the influence of visual content, particularly images and videos, on social media users’ sentiments towards specific sociopolitical events is relatively new but rapidly expanding [1]. Several studies have explored the relationship between visual content and public opinion, with a particular focus on political campaigns, social movements, and crisis events [2–4].

The traditional application of social media sentiment analysis relies on understanding public opinion by employing text-based examination of tweets, comments and posts. The VADER sentiment analysis tool stands out as one of the prominent tools for social media text analysis owing to both its practical design and powerful performance [5]. The exploration of visual content impact on user sentiment remains largely unexamined particularly when studying sociopolitical events.

Data from visual sentiment analysis indicates social media images trigger rapid emotional responses that powerfully shape how the audience feel [1]. In this study, the visual content demonstrate its ability to form narratives which shape the public perception during emergency situations. Previous studies which concentrated on disaster images though the sociopolitical domain introduced distinctive analysis-specific challenges alongside distinct opportunities.

The BERT model together with Transformer-based systems transformed sentiment analysis by delivering text representations that use contextual information [6]. BERT proved valuable for dealing with various sentiment analysis problems, including movie review evaluations alongside social media emotion detection by delivering superior benchmark test results. However, the combination of textual and visual data for sociopolitical sentiment analysis has not received sufficient research attention so far.

One of the key debates in this field concerns the role of visual content in shaping public opinion and sentiment [7]. Some studies suggest that visual content, particularly images, can have a strong emotional impact on users, and thus can significantly shape their attitudes and perceptions towards sociopolitical events [8,9]. Other studies, however, argue that the impact of visual content is limited, as users tend to selectively process information that confirms their existing beliefs and attitudes [10].

Another area of debate concerns the differences between the impact of images and videos on users’ emotions and attitudes. Some studies suggest that videos are more effective in eliciting emotional responses from users compared to images, as they offer a more immersive and dynamic experience [11]. However, other studies suggest that images can have a more significant impact on users, as they are easier to process and can convey complex messages in a concise and effective manner [12].

Furthermore, research has highlighted the importance of social media platforms in shaping users’ sentiments towards sociopolitical events [13–15]. Different social media platforms have different user demographics and content structures, which can impact the way visual content is received and processed [16]. For instance, Instagram is primarily a visual platform, while Twitter is more text-oriented, and Facebook combines both text and visual content.

Overall, the field of research on the influence of visual content on social media users’ sentiments towards sociopolitical events is an evolving and complex area. While there is evidence to suggest that visual content can have a significant impact on public opinion and sentiment, further research is needed to better understand the mechanisms through which this occurs and to identify effective strategies for engaging and informing the public on important social and political issues.

## 3 The methodology

### 3.1 Sociopolitical events

A sociopolitical event refers to any occurrence or incident that has significant social and political ramifications [17–20]. While significant structural transformations are one type of sociopolitical event, events may also be constitutive of social reproduction and continuity. Events can be categorized as either iterative or pulsars [21]. Iterative events are regularly occurring celebrations that tend to confirm social structures [19,22]. In contrast, pulsar events have the potential to transform social structures [23,24]. Events can be seen as social actors that have important influences on social systems, particularly in linking localized small world networks with the global space of flows.

Event system theory suggests that events become salient when they are novel, disruptive, and critical, and that their effects can remain within a single level or travel up or down throughout an organization, changing or creating new behaviors, features, and events [23,25]. Moreover, the impact of events can extend over time as their strength evolves [19,26,27].

In this research, four important movements are considered as examples of sociopolitical events: the Black Lives Matter movement, the Women’s March, climate change protests, and Anti-war demonstrations. These movements are selected for several reasons. First, they address a range of pressing social, political, economic, and environmental issues, deploying diverse tactics and strategies, and enacting demands at multiple scales from local to global levels [28–31]. Additionally, over the past decades, research has consistently shown that these movements are frequently included among the top movements worldwide.

Furthermore, the nature of these topics ensures a broad range of data is available on social media, making them ideal subjects for sentiment analysis and public opinion studies. These movements also demonstrate significant engagement and mobilization on social media platforms, providing rich datasets for examining how visual content influences public sentiment. The Black Lives Matter movement, for instance, has been pivotal in discussing racial justice and police reform, generating substantial online discourse. Chang *et al*. [32] conducted an analysis using real-time scraping during the peak of the BLM movement to capture immediate public sentiment, illustrating the importance of temporal alignment in data-driven studies. Our findings on BLM sentiment trends reflect similar patterns of emotional intensity and public mobilization, even in retrospectively collected data. The Women’s March highlights gender equality and women’s rights, sparking global conversations and widespread social media participation. Climate change protests focus on urgent environmental issues, involving diverse demographics and fostering international dialogue. Anti-war demonstrations address critical issues of peace and conflict, often generating strong emotional responses and substantial discourse on social media platforms.

Moreover, these movements are characterized by their ability to maintain sustained attention and provoke emotional responses, making them suitable for studying the impact of visual content on user sentiments. Their global reach and resonance with various communities ensure diverse perspectives and reactions, enhancing the robustness of the analysis. By examining these movements, this research aims to provide a comprehensive understanding of how visual content on social media shapes public opinion and contributes to the broader discourse on contemporary sociopolitical issues.

Finally, a wide range of social movements targeting racial justice, gender equity, peace and environmental activism are represented by the chosen events, which include the Women’s March, Black Lives Matter, anti-war protests, and climate change protests. In order to ensure that the dataset represents a broad spectrum of public viewpoints and emotional responses, these movements were selected based on their worldwide importance and resonance with diverse communities. The study captures the dynamics of visual content influencing public emotion across many sociopolitical subjects by concentrating on such events together with their motivations and consequences.

### 3.2 Extracting keywords and hashtags

To analyze the impact of visual content on users’ sentiments towards sociopolitical events on social media, relevant keywords and hashtags associated with each event need to be extracted. This involves identifying commonly used words and phrases, including hashtags, slogans, and related keywords, to better understand the conversations and narratives surrounding each event on social media.

The data collection process involves searching for relevant keywords and hashtags on social media platforms like Instagram and Twitter and analyzing posts and comments that contain these terms. The extracted data can then be used to generate word clouds and understand the most frequently used words and phrases, as well as the most popular hashtags and accounts. This information is essential to analyze the content and sentiment associated with each event and evaluate the impact of visual content on users’ emotions and attitudes towards these events.

For this work, in order to cover a comprehensive analysis of a computational social science study, related keywords and hashtags were extracted for each movement from the provided sources, resulting in the top five hashtags for each movement.

**Black Lives Matter:** The most prominent hashtag is #BlackLivesMatter, encapsulating the core message of the movement. The abbreviation #BLM is widely used due to its brevity. #SayTheirNames honors victims of racial violence, while #NoJusticeNoPeace reflects the demand for accountability. #EndPoliceBrutality directly addresses law enforcement reform.

**Women’s March:** The hashtag #WomensMarch unifies all events and messaging. #HearOurVoice amplifies collective demands, #WhyIMarch offers personal motivations, #TimesUp connects to broader gender equity movements, and #WomensRightsAreHumanRights asserts the universality of women’s rights.

**Climate Change Protests:** #ClimateStrike and #FridaysForFuture are directly linked to youth climate activism. #ClimateAction and #ActOnClimate emphasize urgency, while #ExtinctionRebellion highlights civil disobedience movements.

**Anti-war Demonstrations:** #AntiWar and #NoWar express opposition to conflict. #PeaceNotWar and #WarIsNotTheAnswer promote peaceful alternatives, while #EndWar is a general call for global demilitarization.

### 3.3 Data extraction: SocialSense dataset

Data extraction is a crucial step in this study, as it enables the collection of extensive social media data related to various sociopolitical events. For this study, the Instagram platform has been selected because Instagram posts have been shown to provide rich insights into user behaviors and preferences across various domains. Moreover, Instagram, among other social media platforms, is capable of serving as both a visual and textual data sharing platform for users. In this study, the images in the posts serve as visual content, while the comments represent users’ emotions. In other words, by conducting sentiment analysis on the comments, we can understand how the visual content affects users’ emotions. Instagram is an appropriate option for this aim because it has a large user base and allows for the collection of diverse and relevant data, making it ideal for analyzing user reactions to visual content.

**Data Collection and Selection Criteria:**

The dataset was collected from publicly available Instagram posts over a period spanning from 2020 to 2024, ensuring that both historical and recent posts with significant engagement were included. Instagram’s ranking algorithm prioritizes posts with the highest impressions, meaning the most viewed and interacted-with posts appear at the top of search results. This approach allowed us to collect posts that had the greatest visibility and impact on users.

To obtain a structured dataset, data collection was conducted between May, 2024, and July, 2024, during which thousands of posts were identified under relevant hashtags. From this pool, we extracted a total of 100 posts, with 20 posts selected per sociopolitical movement (Black Lives Matter, Women’s March, Climate Change Protests, and Anti-war Demonstrations).

**Post Selection Process:**

Since Instagram prioritizes high-engagement content, the top-ranking posts for each hashtag were reviewed, and the 20 posts per movement were chosen based on the following criteria: High Engagement—posts with the most likes, comments, and shares were prioritized, ensuring they had a significant audience reach. Relevance to the Movement—each post had to directly reference the sociopolitical event through its visual or textual content. Posts unrelated to activism or political discourse were excluded. Diverse Sentiment Representation—posts were selected to include both positive and negative sentiment variations to ensure balanced data distribution. Content-Type Consistency—only posts containing static images (rather than videos or reels) were included to maintain consistency in sentiment analysis methodology.

**Hashtag-Based Data Filtering:**

The research team selected hashtags and keywords to extract data with high diversity and relevance for every sociopolitical event. This process involved:

(a) Relevance to the Movement: The analysis started by selecting primary hashtags after observing their prevalence within sociopolitical movements being investigated. #BlackLivesMatter serves as the movement’s core identifier, and #SayTheirNames added value because it focuses on remembering victims of racial violence.

(b) Popularity: In this research, hashtags that displayed strong popularity and user engagement on Instagram were selected to extract meaningful data from dynamic discussions.

(c) Specificity and Generality: Combining general and specific hashtags such as #ClimateStrike with #ClimateAction served to create a complete perspective of the discussed content.

**Comment Selection and Sentiment Classification:**

Each post contained a varying number of comments. To ensure a manageable yet representative dataset, only posts with 10 to 100 comments were considered. For posts exceeding 100 comments, only the top 100 comments were extracted. This threshold was set to maintain a balance between data volume and processing feasibility while preserving the most relevant user interactions. Sentiment classification was performed using our fine-tuned BERT model, which categorized each comment as either positive or negative, facilitating correlation analysis with the associated image.

**Ethical Compliance and Data Privacy:**

All collected data were obtained from publicly available Instagram posts, adhering strictly to Instagram’s terms and conditions for data usage. No private or restricted content was accessed, and no personally identifiable information was included in the dataset. The study was conducted in accordance with ethical research guidelines, ensuring data anonymization and compliance with social media privacy policies. Additionally, since sentiment classification was conducted at the aggregate level (without tracking individual users), the research does not involve any privacy risks.

A post extraction through the website https://inflact.com delivered content featuring the chosen hashtags. Through this tool, researchers gained access to photos along with their corresponding comments from public Instagram posts. The chosen methodology enabled researchers to obtain a varied and properly representative sample set. The selected study of Instagram worked because it offers user involvement combined with visual and written data collections. The platform matched its functionality well to the specific study of the image-material relationship with user emotions throughout sociopolitical events. This web-based tool allowed access to public Instagram posts, including images and top-level comments, based on specific hashtags. To structure the dataset, we employed a set of Python-based tools. The scraping process was conducted using a combination of the requests and BeautifulSoup libraries to handle HTML parsing and content extraction, while the pandas library was used to organize and store the data in tabular format. These tools enabled automated processing of multiple hashtags and consistent formatting across the different sociopolitical categories.

### 3.4 Data cleaning and categorization

Data cleaning and categorization are fundamental steps in preparing the dataset for analysis. The process involves removing irrelevant or erroneous data, ensuring consistency, and organizing the data into meaningful categories. This step is crucial for obtaining accurate and reliable results from sentiment analysis and understanding the impact of visual content on user sentiments towards sociopolitical events.

Firstly, the data was preprocessed to remove any duplicate entries and irrelevant content. Duplicate posts or comments, spam, and advertisements were identified and excluded from the dataset. This ensured that the dataset only contained unique and relevant social media posts and comments related to the sociopolitical events being studied. Additionally, posts with excessive or unrelated hashtags that did not pertain to the primary sociopolitical event were filtered out to maintain the focus and relevance of the dataset.

The dataset was then categorized based on the specific sociopolitical events and associated hashtags. This categorization helped in structuring the data for more detailed analysis. Each post and its corresponding comments were tagged with the relevant event category, such as “Black Lives Matter,” “Women’s March,” “Climate Change Protests,” or “Anti-war Demonstrations.” Within each category, further sub-categorization was done based on the specific hashtags used, allowing for a granular analysis of different aspects of the events and their related discussions.

To facilitate the sentiment analysis, the textual data (comments) were preprocessed to remove noise and standardize the text. This involved several steps, including: Tokenization—breaking down the text into individual words or tokens. Lowercasing—converting all text to lowercase to ensure uniformity. Removing Stop Words—eliminating common stop words (e.g., “the,” “and,” “is”) that do not contribute significantly to the sentiment analysis. Stemming and Lemmatization—reducing words to their root forms to account for different word variations. Punctuation and Special Characters Removal—removing punctuation marks and special characters that do not add value to the analysis.

Finally, the visual content (images) was categorized based on their themes and characteristics. This categorization was performed by analyzing the visual features of the images, with human attention to replicate the effect of visual content on humans. Images were grouped into categories of positive and negative. By meticulously cleaning and categorizing the data, the study ensured that the dataset was comprehensive, relevant, and structured, laying a strong foundation for the subsequent sentiment analysis and exploration of how visual content influences public opinion on sociopolitical events.

### 3.5 Deep learning model

This research employs a deep learning approach to perform sentiment analysis on social media comments associated with sociopolitical events. The model leverages a pre-trained Bidirectional Encoder Representations from Transformers (BERT) model, fine-tuned for the specific task of classifying sentiments as positive or negative. This section outlines the methodology, including data preparation, model training, evaluation, and the specific components used in the approach.

#### 3.5.1 BERT architecture.

The BERT (Bidirectional Encoder Representations from Transformers) model is a state-of-the-art NLP model developed by Google. It is designed to pre-train deep bidirectional representations by jointly conditioning on both left and right context in all layers. This architecture allows BERT to understand the context of a word based on its surrounding words, providing a nuanced understanding of language.

**Bidirectional Encoding**

Unlike traditional models that read text either left-to-right or right-to-left, BERT reads in both directions simultaneously. This bidirectional approach allows the model to have a deeper sense of context and meaning for each word.

**Transformer Layers**

BERT uses multiple layers of Transformer encoders, each consisting of self-attention mechanisms and feedforward neural networks. The attention mechanism helps the model focus on relevant parts of the input text, enhancing its ability to understand context and relationships.

**Pre-training Tasks**

BERT is pre-trained on two tasks: Masked Language Model (MLM) and Next Sentence Prediction (NSP). MLM involves masking some words in the input text and training the model to predict them, while NSP involves predicting whether two sentences follow each other, improving BERT’s understanding of sentence relationships.

These features make BERT highly effective for a wide range of NLP tasks, including sentiment analysis.

#### 3.5.2 IMDb dataset.

The IMDb dataset is a widely used benchmark for sentiment analysis. It consists of movie reviews labeled as positive or negative, providing a balanced dataset for training and evaluating sentiment analysis models.

**Dataset Composition**

The IMDb dataset contains 50,000 movie reviews, split evenly between positive and negative sentiments. This balanced composition ensures that the model can learn to distinguish between positive and negative sentiments effectively.

**Text Length and Variety**

The reviews in the dataset vary in length and content, offering a rich variety of linguistic expressions and sentiment indicators. This diversity is beneficial for training robust sentiment analysis models that can generalize well to different types of textual data.

**Benchmark for Sentiment Analysis**

Due to its size, balance, and variety, the IMDb dataset serves as an excellent benchmark for evaluating the performance of sentiment analysis models. Models trained on this dataset can be tested for their ability to handle real-world sentiment classification tasks.

#### 3.5.3 Data preparation and tokenization.

The initial step in the model’s workflow involved preparing and tokenizing the dataset. The textual data was preprocessed to ensure consistency and remove noise. This included tokenizing the text into individual words or tokens, converting all text to lowercase, and removing common stop words. Stemming and lemmatization techniques were applied to reduce words to their root forms, and punctuation and special characters were eliminated.

A tokenizer from the Hugging Face Transformers library was employed to transform the comments into a format suitable for input into the BERT model. The tokenizer converted the text into token IDs and generated attention masks, ensuring that each sequence was of a fixed length, which is crucial for maintaining consistency across the dataset.

#### 3.5.4 Model architecture and training.

The BERT model, pre-trained on a large corpus of English text, was fine-tuned on the sentiment analysis task. The model architecture is based on a multi-layer bidirectional Transformer encoder, capable of capturing complex patterns and contextual information in text.

Training the model involved several key steps:

**Dataset Splitting**

The dataset was divided into training and evaluation subsets to ensure that the model’s performance could be reliably assessed.

**Training Configuration** The training process was configured with specific parameters, including the number of epochs, batch size, learning rate, and weight decay. These parameters were optimized to balance training speed and model performance.

**Fine-Tuning**

The model was fine-tuned on the training subset, with the loss function optimized for binary classification. During fine-tuning, the model’s parameters were adjusted to minimize the classification error on the training data.

#### 3.5.5 Evaluation and results.

The model’s performance was evaluated on the test subset, which was not seen by the model during training. Key evaluation metrics included accuracy, precision, recall, and F1-score. The model demonstrated a robust ability to classify sentiments, achieving high performance across these metrics.

#### 3.5.6 Sentiment classification of social media comments.

Following the fine-tuning and evaluation phases, the trained model was deployed to classify sentiments in a large corpus of social media comments. The comments were associated with posts containing specific hashtags related to the sociopolitical events under study.

The classification process involved:

**Batch Processing**

Comments were processed in batches to manage memory usage efficiently. Each batch of comments was tokenized, and the model predicted the sentiment for each comment.

**Sentiment Labels**

The model output logits were converted into sentiment labels (positive or negative) based on the highest predicted probability (Table 1).

**Table 1:** Example comments from the dataset with their predicted labels.

Category	Comment	Label
Black Lives Matter movement	So cute, Adorable	Positive
Black Lives Matter movement	I wonder where these people are now	Negative
Black Lives Matter movement	Justice must be served for all victims.	Positive
Women’s March	Yes please! Need some recharging.	Positive
Women’s March	It’s got nothing to do with being a woman.	Negative
Women’s March	Women’s rights should never be questioned.	Positive
Climate change protests	Good to see parents standing up together to end the fossil fuel era	Positive
Climate change protests	The world is near to its end!	Negative
Climate change protests	Sustainability is key to our future survival.	Positive
Anti-war demonstrations	Peace is the only solution	Positive
Anti-war demonstrations	War is inevitable	Negative
Anti-war demonstrations	History repeats itself, but we must break the cycle.	Negative

#### 3.5.7 Application and insights.

The sentiment classification results were integrated into the broader analysis of how visual content on social media influences public sentiment toward sociopolitical events. By associating the classified sentiments with their corresponding images, the study provided valuable insights into the role of visual media in shaping public opinion.

The training parameters and their significance are summarized in Table 2. These parameters were chosen based on best practices for fine-tuning transformer models.

**Table 2:** Fine-tuning and hyperparameter details for the BERT model used in this study, along with the rationale behind each parameter.

Parameter	Value	Description	Rationale/Significance
Model	bert-base-uncased	Pre-trained BERT model used for sequence classification tasks.	Selected for its state-of-the-art performance in NLP tasks and ability to effectively handle contextual data.
Learning Rate	$5\times 10^{-5}$	Learning rate controls how much the model weights are updated at each step.	Tested with values ranging from $1\times 10^{-5}$ to $1\times 10^{-4}$; $5\times 10^{-5}$ provided the best balance between convergence speed and stability.
Warmup Steps	500	Number of initial steps to stabilize the learning rate during training.	Prevents sudden learning rate changes, tested between 0 and 1,000 steps; 500 steps consistently stabilized training.
Weight Decay	0.01	Regularization parameter to prevent overfitting by penalizing large weights.	Explored values from 0.01 to 0.1; 0.01 was optimal for generalizing well on unseen data.
Batch Size (Training)	8	Number of samples processed per training iteration.	Compared batch sizes of 4, 8, and 16; 8 achieved a balance between memory usage and model performance.
Batch Size (Eval)	8	Number of samples processed per evaluation iteration.	Matched the training batch size to ensure consistent evaluation.
Number of Epochs	3	Total number of passes through the dataset during training.	Increasing beyond 3 led to overfitting, while fewer epochs underutilized the training data.
Evaluation Strategy	Per epoch	Model was evaluated at the end of each epoch to track performance metrics.	Ensured continuous monitoring of overfitting or underfitting.
Optimizer	AdamW	Optimization algorithm designed for transformer-based architectures.	Balanced gradient updates while incorporating weight decay for better generalization.
Logging Steps	10	Training progress was logged every 10 steps for tracking and debugging.	Allowed for visualization of training loss trends and faster troubleshooting.

## 4 Experimental results and computational social study

### 4.1 Calculation of positive percentage for each post through its comments

To understand the sentiment dynamics of each post, we first calculated the positive sentiment percentage for the comments associated with each post. This process involved classifying each comment as positive or negative using our fine-tuned BERT model. For each post, the number of positive comments was divided by the total number of comments, yielding a positive sentiment percentage. This metric provided a nuanced understanding of the sentiment distribution within the user-generated content associated with each visual post.

### 4.2 Sentiment analysis of images

For the visual content, each image was categorized as either positive or negative based on a manual labeling process. Positive images were assigned a value of 100, and negative images were assigned a value of 0. This binary classification enabled a straightforward quantification of the sentiment associated with visual content. This score was then compared with the positive sentiment percentages of the comments to investigate any correlations.

### 4.3 Correlation metrics: PLCC and SROCC

To evaluate the relationship between visual content and user sentiment expressed in comments, we employed two correlation metrics: Pearson Linear Correlation Coefficient (PLCC) and Spearman Rank Order Correlation Coefficient (SROCC). PLCC measures the strength and direction of the linear relationship between two variables, while SROCC assesses the rank-order correlation, making it more robust to non-linear relationships and outliers.

In our study, PLCC was used to determine the linear correlation between the sentiment scores of images and the positive sentiment percentages of the comments. SROCC was applied to assess the consistency of these relationships across different ranks. High values of PLCC and SROCC in our results indicated a strong correlation between the sentiment conveyed by visual content and the sentiment expressed in the associated comments. This finding underscores the significant impact of visual content on public sentiment in social media discussions. Results can be found in Table 3.

**Table 3:** Correlation metrics (PLCC and SROCC) for different sociopolitical events and overall.

	Black Lives Matter	Women’s March	Climate Change Protests	Anti-war Demonstrations	Overall
PLCC	0.615	0.644	0.531	0.709	0.625
SROCC	0.678	0.682	0.611	0.723	0.674

To provide insights into how the BERT-based sentiment analysis model classifies user comments, we present sample predictions in Table 4. Each row shows a user comment, the predicted sentiment, and the confidence score assigned by the model.

**Table 4:** Example comments with BERT-based sentiment predictions and confidence scores.

Category	Comment	Prediction	Confidence
Black Lives Matter	Justice must be served for all victims.	Positive	88.5%
Black Lives Matter	This is all media propaganda!	Negative	87.0%
Women’s March	Empowering to see so many people united!	Positive	89.5%
Women’s March	Feminism is overrated.	Negative	85.2%
Climate Change	If we don’t act now, we are doomed.	Negative	82.0%
Climate Change	Hopeful that leaders will take action.	Positive	83.7%
Anti-war Protest	War only leads to destruction and suffering.	Negative	97.0%
Anti-war Protest	We need peace, not war!	Positive	96.3%

To better illustrate the correlation between visual sentiment and user sentiment expressed in the comments, we present heatmaps and scatterplots showing sentiment distributions across different sociopolitical events.

**Fig 1:**
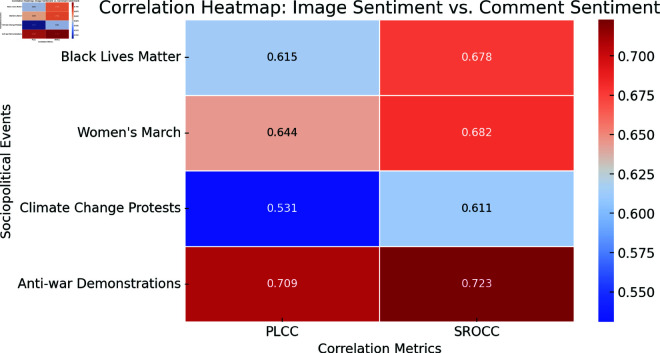
Correlation heatmap showing PLCC and SROCC values for different sociopolitical events.

Fig 1 shows a heatmap where darker colors indicate stronger correlations between the image sentiment and the comment sentiment for each movement.

**Fig 2:**
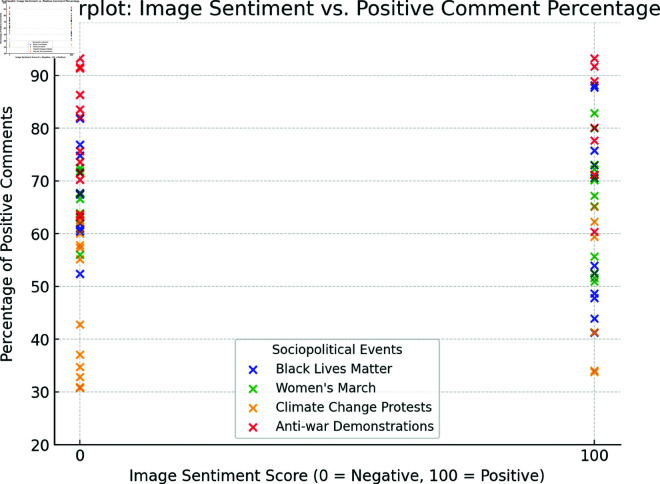
Scatterplots showing the relationship between image sentiment and positive comment percentage.

Fig 2 presents scatterplots for each movement, plotting the image sentiment score (binary: positive = 100, negative = 0) against the percentage of positive comments.

### 4.4 Analysis of study and results

As shown in Table 3, the high correlation metrics from our study suggest that visual content on social media plays a critical role in shaping public sentiment towards sociopolitical events. The positive sentiment percentages for comments and the sentiment scores for images consistently showed significant alignment, indicating that users’ emotional responses to visual content are strongly mirrored in their textual comments. This alignment highlights the influential power of images in communicating and reinforcing emotional narratives on social media platforms.

The analysis further revealed that sociopolitical events characterized by highly emotional visual content, such as the Black Lives Matter movement, climate change protests, and Anti-war demonstrations, exhibited stronger correlations. This suggests that the emotional intensity of visual content can amplify its impact on public sentiment. Additionally, events with a high degree of user engagement and mobilization on social media, such as the Women’s March, also demonstrated strong correlations, emphasizing the role of active participation in shaping public opinion. The Anti-war demonstrations, characterized by their strong visual and emotional content, further underscore the significant role that visual media plays in influencing public sentiment on social media.

### 4.5 Inter-event comparisons of visual influence

This research sought to examine different movements by analyzing shifts in correlation metric values (PLCC and SROCC) to understand how visual media impacts changes over time. The results indicate that Anti-war Demonstrations exhibit the highest correlation (PLCC: 0.709, SROCC: 0.723). Visual compounds related to war events demonstrate the strongest link between visual effect and linguistic sentiment throughout the studied movements.

Because of their impact on human brain, images of conflict frequently elicit strong emotional reactions right away. According to psychological and cognitive science research, pictures that depict violent incidents, destructive devastation, or suffering elicit strong emotional reaction far more quickly compared to abstract social issues. According to empirical research, images from anti-war protests typically feature battle casualties alongside damaged infrastructure and non-violent activism, which reveal strong emotional reactions from those who favor peace initiatives or theological viewpoints.

The correlation for Climate Change Protests, on the other hand, is smaller (PLCC: 0.531, SROCC: 0.611). Images pertaining to climate had lower sentiment scores, most likely because further context is necessary to fully understand their impact on urgency. Without accompanying written content, a landscape painting of freezing ice or deforestation might not elicit strong emotional reactions, yet images of war instantly evoke strong emotions in everyone due to their acknowledged intrinsic ability to evoke feelings. These emotions may be revealed differently by by different people based on how they envision the rights of each party involved in the conflict. In some cases, it is even possible to witness people supporting the killing of others based on own norms, political standing, etc.

There are mid-range connections between the Black Lives Matter and Women’s March movements (PLCC: 0.615 and 0.644, respectively). These motions’ wide range of visual material elicits a range of emotional reactions which contribute to their intermediate PLCC scores. The diverse variety of iconography of the social justice movement elicits a wide range of reactions from people whose cultural, religious, and ethnic backgrounds influence their views.

Key findings on the role of visual content in different events are listed below.

(a) Events with explicit and emotionally charged visuals (e.g., war, crisis) show the highest correlation between visual and textual sentiment.

(b) Movements reliant on symbolic imagery (e.g., Climate Change Protests) show lower correlation, as their impact often depends on prior knowledge and contextual framing.

(c) Protests featuring diverse messaging (e.g., Black Lives Matter, Women’s March) tend to have moderate correlation values due to variations in image types and interpretations.

This study shows that the influence of visual media on feelings depends on the social and political environment as well as the attributes of the image. The findings suggest that, while social issue movements exhibit a range of emotional responses, direct human suffering movements (such as those involving war and police brutality) provide regular alignment between image-emotion pairs.

### 4.6 Further discussion and future improvements

Practical Implications:

This study reveals useful information which supports practical applications for different groups of users. The results provide key knowledge for lawmakers who need to create new regulations which ensure proper ethical practice along with effective AI content moderation solutions. Such regulations must ensure transparency while striving for fairness because this combination helps in reducing both technical and harmful aspects of automated systems.

Social media platforms will have a solid option to improve their current content screening solutions by implementing this model. Through its context-based toxic language detection mechanism the model enhances accurate harmful content identification while reducing incorrect false alarms. When implemented correctly, these models can improve platform usability and generate safer internet experiences for users.

The results from this study help advocacy groups create stronger public understanding about toxic language effects in the society. The knowledge acquired through this study enables advocacy organizations to create educational materials and resources which promote respectful digital communication for users online. The specific efforts show great potential to address the issues of online harassment together with hate speech.

While our results are promising, several aspects of the study can be improved to enhance the realism and robustness of the findings. One limitation is the manual labeling process for images, which can introduce subjectivity. Future studies could incorporate a broad range of subject to label the visual contents in more fair manner.

Moreover, the current binary classification of images as either positive or negative may oversimplify the complexity of visual content. Incorporating a multi-class classification approach that considers a spectrum of sentiments (e.g., very positive, somewhat positive, neutral, somewhat negative, very negative) could provide a more detailed understanding of the sentiment landscape.

Another area for improvement is the diversity of data sources. While this study focused on Instagram, expanding the analysis to include other social media platforms such as Twitter, Facebook, and TikTok could provide a more comprehensive view of public sentiment across different user demographics and content formats.

In addition to textual and overall visual content analysis, future research may benefit from disambiguating sentiment at a finer level by incorporating facial feature analysis from the individuals depicted in social media images. Recent work by Wei *et al*. [33] demonstrates that facial expressions can provide a richer and often divergent emotional context compared to accompanying text, particularly in high-stakes political environments. Although this method was not incorporated in our current study, it offers a compelling direction for future research aiming to deepen the granularity and interpretability of multimodal sentiment dynamics in sociopolitical discourse.

Finally, integrating contextual factors such as the timing of posts, the presence of influential users, and the virality of specific content could offer deeper insights into the dynamics of sentiment formation. Understanding these factors could help in identifying the key drivers of public opinion and developing more effective strategies for sociopolitical communication on social media. Future research should analyze bigger data collections while studying political attitudes using both Twitter and TikTok social media networks together and studying the extended effects of visual content. The research establishes fundamental knowledge about these dynamics to advance exploration of the intricate social media-public sentiment-sociopolitical movement connections within contemporary digital platforms.

This study has one major limitation that there may exist AI generated or synthetic images in the dataset. Our dataset was also collected retrospectively by way of high engagement ranking algorithms that might potentially include images reflective of public sentiment, thematic alignment rather than true authentic temporal authenticity. It exposes the risk of making the appearance of the organic visual meaning of the time with misrepresentation. Chang *et al*. [34] has also emphasised the notion of visual authenticity in research on social media and are found to be aligned with the notion that visual narratives on war draw the most emotional impact downstream. Such findings are consistent with our observation that anti-war imagery had the strongest sentiment correlation. Going forward, it is advisable to develop the technology for automatically detecting AI generated content and filtering datasets accordingly so that we maintain historical fidelity in sociopolitical analysis.

## 5 Conclusions

The outcome from this research shows that visual content strongly influence how social media users react to sociopolitical events. Our research using deep learning-based sentiment analysis of Black Lives Matter Instagram posts alongside Women’s March content and Climate Change Protests and Anti-war Demonstrations content demonstrate a solid connection between public emotion and visual content. Analysis results show diverse levels of agreement between the public opinion and different sociopolitical occurrences. Anti-war Demonstrations exhibited the strongest correlation (PLCC: 0.709, SROCC: 0.723). Public sentiment responds most intensely to conflict-related images because of their emotionally charged nature. In contrast, Climate Change Protests had the weakest correlation (PLCC: 0.531, SROCC: 0.611). The results suggest that emotional imagery might become less effective for climate change protests due to the dense scientific nature of environmental activity discourse. The response from the public demonstrates unique engagement dynamics toward different events depending on which visual elements appear. Based on the examined dataset, these research findings establish important consequences regarding sociopolitical engagement in the digital age. These days, social media platforms serve as venues for people to develop political movements and to establish and influence the public opinion. Understanding how strong emotions are evoked by vivid images helps activists in creating visually striking tactics that increase awareness and strengthen movement support. Because visual content can evoke strong feelings, there are concerns that unreliable information may be disseminated online along with purposefully distorted opinions and manipulated feelings. The knowledge about visual narrative power enables policymakers and both activists and researchers to develop better communication methods and challenge misinformation while analyzing public opinions. These findings advance both computational social science investigation and analytic methods for social media platforms by showing real-world evidence about photo-based social media’s impact on public emotions across varied sociopolitical climates.

## Appendix A: Temporal distribution of collected instagram posts

**Fig 3:**
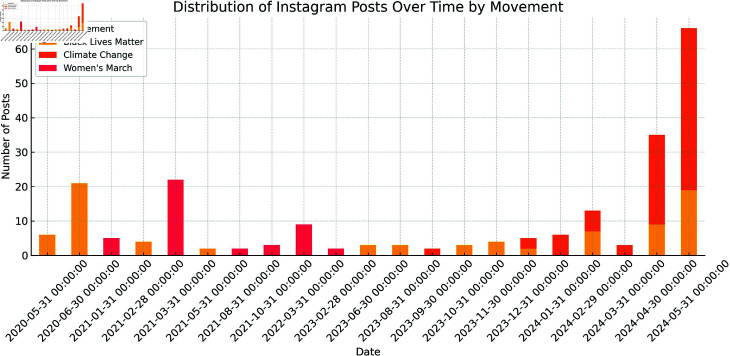
Distribution of collected Instagram post timestamps by sociopolitical movement.

To address potential recency bias in the dataset, we analyzed the timestamp distribution of all collected Instagram posts. Fig 3 shows the time series of post dates, indicating that our scraping approach—based on Instagram’s engagement-based ranking algorithm—resulted in a broad temporal range of posts, including posts from 2020 to 2024. This supports our claim that although the scraping process began in May 2024, the dataset captures earlier influential content as well.
